# Measures of Compositional Strand Bias Related to Replication Machinery and its Applications

**DOI:** 10.2174/138920212799034749

**Published:** 2012-03

**Authors:** Kazuharu Arakawa, Masaru Tomita

**Affiliations:** Institute for Advanced Biosciences, Keio University, Fujisawa 252-8520, Japan

**Keywords:** Nucleotide composition bias, bacterial replication, GC skew, replication-related mutations.

## Abstract

The compositional asymmetry of complementary bases in nucleotide sequences implies the existence of a mutational or selectional bias in the two strands of the DNA duplex, which is commonly shaped by strand-specific mechanisms in transcription or replication. Such strand bias in genomes, frequently visualized by GC skew graphs, is used for the computational prediction of transcription start sites and replication origins, as well as for comparative evolutionary genomics studies. The use of measures of compositional strand bias in order to quantify the degree of strand asymmetry is crucial, as it is the basis for determining the applicability of compositional analysis and comparing the strength of the mutational bias in different biological machineries in various species. Here, we review the measures of strand bias that have been proposed to date, including the ∆GC skew, the B_1_ index, the predictability score of linear discriminant analysis for gene orientation, the signal-to-noise ratio of the oligonucleotide bias, and the GC skew index. These measures have been predominantly designed for and applied to the analysis of replication-related mutational processes in prokaryotes, but we also give research examples in eukaryotes.

## INTRODUCTION

Genomic nucleotide sequences exhibit extraordinary variability in their guanine + cytosine (G + C) compositions, ranging from as low as 16.6% in *Carsonella ruddii* [1] and up to 74.9% in *Anaeromyxobacter dehalogenans* [2]. While the cause for this plasticity is still debated, proposed reasons include energetic [3, 4], genetic [5], biochemical [6], and environmental factors [7, 8] (see [2] for a recent review). Almost all of the genomes sequenced to date show symmetry in the composition of complementary bases: A ≈ T and G ≈ C. This intriguing equilibrium of the complementary bases within a single strand of the DNA duplex is commonly referred to as the Chargaff’s second parity rule (PR2) [9]. Chargaff’s first parity rule of complementary bases in the duplex DNA molecule [10] defines the base pairing in the Watson-Crick model [11], and Chargaff and coworkers later extended this rule to a single strand of the duplex based on empirical observations [12]. Using hundreds of complete genomes, extensive computational analysis recently showed that PR2 applies to almost all double-stranded DNA genomes that are sufficiently long [13]. Sueoka showed that PR2 is also expected to theoretically hold under no-strand-bias conditions, where the mutation rates are similar between the two strands [14].

PR2, however, is violated when the mutation/selection rates in the two strands of the DNA molecule are not at equilibrium. This would occur, for example, in single-stranded viral genomes and in organellar genomes, such as mitochondria, that harbor a unidirectional mode of replication, where the mutation rates are asymmetric in the two strands [13, 15]. In fact, such deviation from PR2 is almost universal in local regions of genomic sequences. Szybalski and coworkers first noted an excess of purines over pyrimidines in the coding sequences of bacteriophage in 1960s [16], and this transcription-coupled compositional asymmetry (presumably due to coding requirements and transcription-coupled repair and mutagenesis) has generally been confirmed in a variety of species [17-19]. In eubacteria with circular chromosome, replication progresses bidirectionally along the genome from a finite origin until the replication forks meet at a terminus [20]. This theta-type of replication results in two replicating arms of equal lengths, or the replichores, with opposite polarity in nucleotide composition due to asymmetric modes of leading and lagging strand synthesis [21]. While the genome as a whole maintains compositional parity because the opposite polarity of the replichores cancel each other out, each replichore shows a highly asymmetric composition of complementary bases, especially characterized by an excess of G over C in the leading strand [21, 22]. Chromosome replication therefore exerts a strand-specific mutation/ selection pressure and results in a number of biases in genomic features in addition to the asymmetric compositional bias [23, 24]. First, coding sequences are preferentially located in the leading strand in most genomes [25], and this gene strand bias is as high as 90% in *Acetohalobium arabaticum* (but there are exceptions [26, 27]). Moreover, base as well as codon composition of genes are also biased depending on the strand [28-32]. Second, a large number of complementary oligonucleotides are asymmetrically distributed in the leading and lagging strands [33]. These oligonucleotides include highly skewed octamers that are overrepresented in *Escherichia coli*, such as the homologous recombination hotspot named the Chi site, which is recognized by the RecBCD recombinase [34-36], and the FtsK-orienting polar sequences (KOPS) that direct FtsK translocase to the *dif* site, where XerCD recombinase binds in order for chromosome dimer resolution *via *homologous recombination [37-39]. Chi-analogues are found in multiple phylums [40], and KOPS-like sequences have been computationally predicted in hundreds of bacteria [41]. Third, strand preference correlates with gene essentiality [42] and lengths [43, 44], and replication further affects gene positioning and the organization of chromosomal domains [45, 46]. While the relationship between the orientation of replication and transcription is still controversial, a similar replication-related strand bias in terms of nucleotide and oligonucleotide composition and gene orientation has also been observed in eukaryotes, including mammals [47-50]. 

A deviation from PR2 in the nucleotide sequence therefore implies the existence of a mutational or selectional bias in the DNA duplex, and consequently, a biological mechanism that generates the strand bias. Therefore, analysis of the shift-points of strand bias can be used to identify transcription start sites in eukaryotes [51-53] and for the prediction of replication origins and termini in prokaryotes [54-56] and eukaryotes [50]. Strand bias is commonly calculated as the relative excess of a base among the complementary bases [22, 57] using the formula

(1) $$\mathrm{XYskew}=\left(X-Y\right)/\left(X+Y\right)$$

where X and Y represent the complementary bases. While the most frequently used combination of *X *and *Y* are C and G (GC skew), or T and A (AT skew), other combinations of bases are also used, such as purines (G and A) and pyrimidines (C and T), as well as keto bases (G and T) and amino bases (AC) [58]. The set of GC skew and AT skew is alternatively called the Chargaff difference, where the variables in the above formula are often replaced by the IUPAC codes for degenerate nucleotides; i.e., S for G + C, and W for A + T [59]. *X* and *Y* could even be genes on the Watson and Crick strands of DNA that could be used to calculate the gene orientation skew. The use of Euclidean distance from a theoretical parity point is also demonstrated as a combination of the AT and GC skews, expressed as [15]:

(2) $$d\left(\mathrm{PR2}\right)=\sqrt{\mathrm{AT}{\mathrm{skew}}^{2}+\mathrm{GC}{\mathrm{skew}}^{2}}$$

Local regions with strand bias are detected by plotting this nucleotide skew along the sequence using sliding windows. Nucleotide skew is close to zero when there is no strand bias; however, when a sequence window contains an excess of Gs over Cs, the GC skew becomes a negative value based on the above formula, (C-G)/(C+G). Because the circular chromosome of eubacteria is subdivided into two replichores of opposite polarity that correspond to the leading and lagging strands, respectively, the GC skew graph of this genome shifts its sign at the junction of the two replichores. These shift-points in the GC skew graph correspond to the replication origin and terminus in eubacteria, and it is the basis for skew-based prediction of these sites (see [60, 61] for lists of software for origin prediction in bacteria). The uses of two-dimensional [62, 63] and three-dimensional [64, 65] trajectories have also been explored in order to accommodate multiple sets of bases and to overcome the need for windowed calculations. Cumulative plots of nucleotide skews are also frequently used in the analysis of skew shift points, which become the maxima and minima in these graphs [66]. Cumulative skew diagrams also correspond to the projection of random walk graphs for a single pair of complementary bases.

Numerous methods have been proposed for the analysis of compositional asymmetries, including multivariate statistics [31, 67] and signal processing methods based on Fourier [68] and wavelet transformations [50, 69] and wavelet-based multifractal analysis [70] (see [61] for comprehensive review). The key to these analyses is the quantitative measurement of the strength of strand bias. The prediction of replication origins by the analysis of compositional asymmetry is used in almost all sequencing projects of circular, bacterial genomes in order to name the first base of the sequences submitted to a public database based on the position of the replication origin. However, skew-based prediction is only applicable when a GC skew is clearly visible in the genome, which is not necessarily true in all prokaryotes [33, 71]. Quantitative indices of the degree of replication-related strand bias within these genomes become useful in such cases to provide the prerequisite validity of the application of skew-based analyses. Moreover, such measures of strand bias are indispensable for comparative studies of strand-specific mutational and selectional biases exerted by various mechanisms and for evolutionary studies of genetic changes in multiple genomes. To this end, we review several measures of replication-related strand bias and their applications in studying compositional asymmetry in genomic sequences.

## MEASURES OF STRAND BIAS

### ∆GC Skew and B_I_ Index

The GC skew is simply the measure of the bias towards a base within a pair of complementary bases; therefore, it consists of the sum of all mutational effects and does not delimit those of replication from transcription. Therefore, the simplest approach to extract only the replication-related strand bias is to take the difference of the GC skew values in the leading and lagging strands, so that other biases that are independent from the replication bias will cancel out. This ∆GC skew was first proposed in one of the earliest studies that used GC skew and is defined as follows [22]:

(3) $$\Delta \mathrm{GCskew}=\mathrm{GCske}w_{\mathrm{leading}}-\mathrm{GCske}w_{\mathrm{lagging}}$$

∆GC skew can be used with other nucleotide pairs, including AT, keto, and amino bases, to calculate the skew. However, the above formula cannot exclude the effects of biased gene distribution in the leading and lagging strands and may contain strand bias effects from coding requirement or transcription. Followed by the work by Mrazek and Karlin that differentiated the effects of replication and transcription [26, 27], Rocha and coworkers therefore proposed the normalized ∆GC skew of genes for this purpose [72]:

(4) $$\Delta \mathrm{GCske}w_{\mathrm{gene}}=\frac{\sum_{{i\in \mathrm{genes}}_{\mathrm{leading}}}^{}{\mathrm{GCskew}}_{i}}{N_{\mathrm{leading}}}-\frac{\sum_{{i\in \mathrm{genes}}_{\mathrm{lagging}}}^{}{\mathrm{GCskew}}_{i}}{N_{\mathrm{lagging}}}$$

They later further simplified the formula by considering only the third codon position of four-fold degenerate codons (*q*) [73] such that

(5) $${\Delta \mathrm{GCskew}}_{q}={\mathrm{GCskew}}_{q,\mathrm{leading}}-{\mathrm{GCskew}}_{q,\mathrm{lagging}}$$

While the ∆GC skew is stronger than the ∆AT skew in almost all bacteria, ∆AT skews are nonetheless observed in a number of species, and is sometimes more represented than ∆GC skew especially in A+T rich genomes [72]. Therefore, an index that takes account of both the GC and AT pairs is proposed by Lobry and Sueoka, as follows [74]

(6) $$\begin{array}{c} B_{I}=\sqrt{{\left(x_{\mathrm{leading}}-x_{\mathrm{lagging}}\right)}^{2}+{\left(y_{\mathrm{leading}}-y_{\mathrm{lagging}}\right)}^{2}}\\ x=\frac{G}{G+C}\\ y=\frac{T}{T+A} \end{array}$$

where *A, T, G, *and *C *are the frequencies of the corresponding nucleotides in the third codon positions. Consequently, the overall contribution of transcription- and translation-related bias can be formulated by the averages of *x* and *y *(*x_c_* and *y_c_*) of the leading and lagging strands:

(7) $$B_{\mathrm{II}}=\sqrt{{\left(x_{c}-0.5\right)}^{2}+{\left(y_{c}-0.5\right)}^{2}}$$

As with the calculation of the ∆GC skew, Rocha and coworkers later modified the B_I _index to only consider four-fold degenerate codons [73]. The maximal value of GC skew is 1 (when only C is found in a sequence), and therefore, the maximum ∆GC skew is 2 and that of B_I_ is $\sqrt{2}$. When there is no strand bias, both indices become 0.

### The Predictability Score of Linear Discriminant Analysis of Gene Orientation

Nucleotide compositional skews often coincide with gene orientation skews [25, 67]. Genes are almost always more likely to be found on the leading strand, and there are presumably mutational or selectional pressures that affect the choice of nucleotides, codons, and amino acids. Linear discriminant analysis (LDA) is one of the simplest multivariate statistics approaches to compare these effects, particularly as there are only two states to be considered with regard to gene orientation, that is, whether a gene is found on the leading or the lagging strand. Here, the linear discriminant function is first obtained from the explanatory variables of interest [31] with

8 $$F\left(x\right)=a_{0}+\sum_{i=1}^{n}\alpha_{i}x_{i}$$

where *α_i_* is the calculated contribution coefficients for *i*th variable having composition *x_i_* such that *F(x)* > 0 describes one group and another group is described by *F(x) *< 0. The number of variables, *n, *is therefore 4 for nucleotides, 12 for nucleotides in each codon position, 61 for codons, and 20 for amino acids. The predictabilities of the obtained discriminant functions can then be compared to assess the relative effects of compositional biases. Moreover, by calculating the predictability score along the genome one gene at a time and by dividing genes into two groups (left or right) relative to the observation position, the replication origin or terminus can be predicted to occur at the position where the predictability is highest. Nucleotides and codons generally have high prediction accuracy; however, the amino acid composition also shows a weaker but still clear predictability [31]. When there is no compositional bias, the prediction accuracy of LDA becomes 50%, implying a completely random distribution of the two classes (leading or lagging). The presence of stronger bias brings this number up to 100% (completely biased).

### The Signal-to-Noise Ratio of Oligonucleotide Bias

The cause for the skew of oligonucleotides is multifactorial, including the Markovian probability based on the nucleotide composition [34, 36], the specific selection of biological signal motifs, such as Chi and KOPS [21, 67], and the codon usage, especially in light of biased gene distribution [73]. Nevertheless, strand bias generally coexists with nucleotide composition and the distribution of complementary oligonucleotides [33]. Worning and coworkers therefore presented the use of the oligonucleotide strand bias for the prediction of the replication origin by calculating the weighted double Kullback-Leibler distance, *D_i_,* of each oligonucleotide, *I,* whose occurrence count is *N_i_* as

(9) $$D_{i}=\left(N_{i,\mathrm{leading}}-N_{i,\mathrm{lagging}}\right)\log_{2}\left(\frac{N_{i,\mathrm{leading}}+r}{N_{i,\mathrm{lagging}}+r}\right)$$

where *r *(=5) is a control number to accommodate the low frequency of oligonucleotides. Here, *N_i,leading_* and *N_i,lagging_* are complementary oligonucleotides in a single-stranded sequence, and therefore, this calculation cannot be applied to palindromic (self-complementary) sequences such as 5’–GATC–3’, which are identical on both strands. This *D_i_* is calculated for all of the applicable oligonucleotides up to octamers, and the sum of all *D_i_* at a position, *p,* is considered the overall bias:

(10) $$S_{p}=\sum_{i\in \mathrm{oligo}}D_{i}$$

*S_p_* is calculated by moving the position, *p,* along the genome, and the regions upstream and downstream of *p* are considered as hypothetical origins. *S_p_* is repeatedly calculated for window sizes of 50%, 55%, 60%, 65%, and 70% of the genome, and the median *S_p_* among these windows is chosen, and is normalized to have the same scale as *S_p_* calculated using the window size of 60% of the genome. Similar to the prediction accuracy of LDA, the *S_p_* should be maximal at the real replication origin. Worning and coworkers took the signal-to-noise ratio of *S_p_* further as the unique measure of strand bias for a given genome by using the maximal *S_max_* and minimal *S_min_*:

(11) $$S/N=\frac{S_{\max}}{S_{\min}}$$

Unlike other measures of strand bias, the S/N of oligomer skew does not have a theoretical maximum value for completely biased sequences (such as 2 for ∆GC skew, 1 for B_I_, 100% for prediction accuracy of LDA, or 1 for GC skew index). When there is no strand bias, the S/N is at its minimum and is equal to 1. 

### The GC Skew Index

The calculation of the ∆GC skew or B_I_ requires the knowledge of the positions of origin and terminus that are typically predicted computationally using the skew shift points. Therefore, the application of these indices for skew analyses could become circular. Moreover, the skew shift points in the GC skew, or the maxima and minima in the cumulative GC skew graphs may not represent the true origin and terminus of replication in weakly biased sequences where genomic inversions and the horizontal transfer of biased sequences can introduce pseudo-shift points. In eubacteria, the replication-related strand asymmetry results in two replichores of nearly equal lengths (note that replichores are not exactly symmetrical in many bacteria, especially in the phylum Firmicutes) but with opposite polarity. GC skew graphs in these species therefore resemble the graph of a discrete sine curve, a graph composed of Y = -1 for t_0_ ~ (t_1_ - t_0_)/2 and Y = 1 for (t_1_ - t_0_)/2 ~ t_1_, and this “shape” of the GC skew graph can be assessed by observing the strength of the 1 Hz signal of its Fourier transformation [68]. Fourier transformation mathematically decomposes a given signal into a set of constituent frequencies, and the most simple frequency component of 1 Hz corresponds to a sine curve spanning all across the given signal duration. Therefore, pattern recognition of the shape of shifting GC skew graph can be mathematically considered as finding the 1 Hz spectral component of a given signal. The GC Skew Index (GCSI) thus combines the strand compositional difference that is calculated like the ∆GC skew (*dist_norm_*) with the conformity of the GC skew graph to a discrete sine wave (*SA*) using a Fast Fourier Transformation (FFT) by taking the geometric mean of the two values [75, 76] such that

(12) $$\mathrm{GCSI}=\sqrt{k_{1}\mathrm{SA}\times k_{2}{\mathrm{dist}}_{\mathrm{norm}}}$$

where *k_1_* and *k_2_* are normalization constants that have been empirically obtained to be 1/6000 and 1/600, respectively, in order to set the range of GCSI to 0 (no bias) or to approximately 1 (high bias). The *SA* is the spectral amplitude of the 1 Hz signal obtained by FFT, *F*(*k*), of a signal of length, *N*, *f*(*n*), where *n* = 0, 1, …, *N* – 1, at the frequency, *k*, is calculated as follows

(13) $$F\left(k\right)=\sum_{n=0}^{N-1}f\left(n\right)e^{-\mathrm{i2}\pi \mathrm{kn}/N}$$

where *i* = $\sqrt{-1}$. The power spectrum, *PS*(*k*) of *F*(*k*), is then given by

(14) $$\mathrm{PS}\left(k\right)={\left|F\left(k\right)\right|}^{2},k=0,1,2,...,N-1$$

at each frequency, *k*. The 1 Hz signal is therefore *PS*(1). The *SA* is calculated by normalizing this signal strength as

15 $$\mathrm{SA}=k_{4}{\left(k_{3}\mathrm{PS}\left(1\right)\right)}^{\alpha }$$

where *k_3_*=600,000, *k_4_*=40, and *α*=0.4, as calculated by regression analysis. The *dist_norm_* is calculated from the ∆GC skew with a windowed calculation using all bases (i.e., not limited to the third codon position) and using the regions between the maxima and the minima of the cumulative GC skew graphs, as follows

(16) $${\mathrm{dist}}_{\mathrm{norm}}=:\Delta \mathrm{GCske}w_{\mathrm{all}}\times 4096/W$$

where *W* is the number of windows used in the analysis for normalization.

Although windowed calculation of GC skew is required to produce initial signal for FFT, the GCSI does not depend on the choice of window size, as long as the number of windows is a power of 2. A window number of 2048 is recommended for eubacteria because each window contains regions longer than approximately 1 Kbp in these genomes (approximately 2 Mbp ~ 4 Mbp in size), which eliminates the local strand bias effects exerted by coding sequences that can be an average of 1 Kbp long. In small genomes, such as plasmids, a window number greater than 32 is recommended. GCSI also provides *P *values for the significance of its value based on *z-*testing with randomized iterations. A GCSI > 0.05 usually implies the existence of a strand bias in genomes with bidirectional replication machinery. 

## IMPLICATIONS OF THE DIFFERENT MEASURES OF STRAND BIAS

The five measures of replication-related strand asymmetry described above are summarized in Table **1**, and these indices can be categorized into two groups based on the requirement for knowledge of the coding regions and the positions of the replication origin and terminus. ∆GC skew, B_I_, and LDA prediction accuracy require such information, and these indices are predominantly used in detailed studies of the mutation pressures that determine the mechanisms that shape the replication-related strand bias [72-74, 77-79]. The mechanism for replication-associated strand asymmetry has frequently been attributed to the cytosine deamination theory [21, 80, 81], which is based on evidence that single-stranded DNA is significantly more vulnerable to the spontaneous hydrolysis of cytosine to uracil, resulting in a C->T transition [82-84]. Due to the discontinuous strand synthesis of Okazaki fragments in the lagging strand, the leading strand is more prone to cytosine deamination, and this type of mutation had been generally accepted as one of the theories for strand-specific mutation. However, the aforementioned series of studies analyzed the mutation patterns using a large number of closely related chromosomes with an extremely careful selection of orthologous genes. By eliminating the effects of selection in combination with the strand bias measures, it is now clear that a C->T transition by cytosine deamination is not the only mutational pressure, and that different clades of bacteria are affected by different mutational biases [73] (see [23, 85, 86] for possible biological mechanisms causing these mutations). To summarize, these indices are designed for the careful analysis of mutational forces that shape the replication-related strand asymmetry and are needed to rule out the effects of selection and other mutational pressures; therefore, each of these indices represents a specific mutational effect. ∆GC skew and B_I _both eliminate the effect of transcription-related mutation, and ∆GC skew only observes the G/C mutation bias whereas B_I_ observes the combined effects on A/T and G/C. LDA is intended for the study of replication-related mutation bias on the nucleotide or codon composition of coding regions, and S/N of oligomer skew observes that for short oligonucleotides. GCSI does not separate these mutational effects, and quantifies the overall sum of replication-related mutations affecting on the genome. 

The other two indices, S/N of oligomer skew and GCSI, do not require *a priori* knowledge of the coding regions or the positions of the replication origin and terminus, and they tend to indicate the overall contribution of different mutational forces, including the effects of nucleotide composition bias and gene orientation bias. Therefore, these measures are useful for assessing the significance of skew-based predictions of replication origins and termini, and to quantify the strength of strand asymmetry for comparative studies among hundreds of genomes. For example, Rocha and coworkers and later Worning and cowerkers showed that the degree of strand bias shows a characteristic distribution in different phyla of eubacteria (reproduced with modifications in Fig. (**1**)) and that the direction of the AT skew is correlated with the presence of the *polC* subunit of DNA polymerase, which results in an asymmetric proofreading system in the leading and lagging strands [27, 87]. The type of DNA polymerase holoenzyme subunit structure is also known to affect the gene strand bias in these genomes [27]. A comparative study using GCSI characterized the different types of replication, such as bidirectional replication in eubacteria, replication from multiple origins in archaea, and the theta and rolling circle methods of replication in bacterial plasmids [75]. Because the GCSI checks the conformity of the GC skew graph to the discrete sine curve that is a characteristic of bidirectional replication mechanisms, the GCSI is low when there are multiple origins of replication, as in archaeal species. Likewise, the rolling circle method of replication results in the entire strand of DNA being a leading strand, which results in a continuously rising cumulative GC skew graph, and subsequently, a high *dist_norm_* and a low *SA*. As a result, the GCSI becomes relatively high, but its *P-*value significance would be low. We have also showed a high correlation between the GCSI of plasmids and their corresponding host chromosomes [75], suggesting that amelioration within cells can be caused by replication-related mutation, in addition to reported amelioration for genomic signatures [88-91]. 

While the objectives of the indices of strand bias are different, as discussed so far, they are generally correlated to each other, as shown in Fig. (**2**) by clustering the indices according to their Pearson correlation using the calculated strand bias values for 1083 bacterial genomes. With the exception of the S/N of oligomer skew with several of the LDA prediction accuracies, almost all of the strand bias indices were highly correlated to each other, with *r* > 0.70. For comparison, the inverse of the doubling time collected from the literature for 254 species [92] and several other features that are related to the bacterial growth rate, including the copy number of tRNAs and rRNA operons as well as the Sharp’s S-index for the strength of selected codon usage bias [93], were also used in the cluster analysis. While the strand bias indices are not as highly correlated to the growth rate as the known features, they nevertheless show a weak correlation (*r *> 0.25). This is consistent with the observations that slow growers, such as Cyanobacteria and *Mycoplasma* species, are known to have a very limited GC skew [76, 87] and that the fast growers tend to have a higher gene orientation bias, such as in Firmicutes, presumably to avoid the head-on collision of DNA and RNA polymerases [94, 95], although other mechanisms have also been suggested [23]. Note however, that the possible existence of multiple origins is also suggested as the reason for unclear GC skew [96]. 

## STRAND BIAS STUDIES IN EUKARYOTES

The initiation of replication is highly diverse in eukaryotes. *Saccharomyces cerevisiae* is known to have highly conserved autonomously replicating sequences (ARSs) of about 200 bp in length [97] and replication-related strand asymmetry is only observed in the subtelomeric regions in this species [47]. The replication origins span a broader region of the chromosomes in *Schizosaccharomyces pombe* [97]. In *Drosophila* and *Xenopus *embryo where the S-phase is extremely rapid [98-100], the replication origins are distributed randomly [101] and any regions of DNA seems to be able to function as a replicator. The initiation sites for replication in mammals are more strictly defined; however, their locations are not encoded in the nucleotide sequence as motifs, but rather, are epigenetically coded by the chromatin organization [102-104]. The availability of experimentally defined origins and more comprehensive origin mapping studies by chromatin immunoprecipitation (ChIP) analysis coupled with microarrays (ChIP on chip) or deep sequencing (ChIP-Seq) is opening the door to a number of computational studies regarding replication-related strand asymmetry in the human genome [105-109], and mechanisms leading to eukaryotic strand bias are getting reported [110, 111].

The study of strand asymmetry around human replication origins primarily uses alterations in the GC skew [19, 48-50, 112-115]:

(17) $$\begin{array}{c} S_{\mathrm{GC}}=\frac{G-C}{G+C}\\ S_{\mathrm{AT}}=\frac{T-A}{T+A}\\ S=S_{\mathrm{TA}}+S_{\mathrm{GC}} \end{array}$$

When considering the regions upstream and downstream of replication origins, the degree of strand asymmetry around these sites can be defined as:

(18) $$\Delta S=S_{\mathrm{upstream}}-S_{\mathrm{downstream}}$$

As with the GC skew analysis, the *S *values are plotted along the nucleotide sequence using a sliding window of 1 Kbp, and cumulative graphs are also used to identify the shift points. Unlike the GC skew in prokaryotes, where the nucleotide composition is uniform across a strand, the *S* in eukaryotes rapidly diminishes and approaches 0 as the distance from the origin of replication increases. This results in an “N-shaped” graph, unlike the GC skew graph of prokaryotes, which resembles a discrete sine curve. Based on this N-shaped *S* graph coupled with wavelet analyses, strand asymmetry is used to predict replication origins and to study the effects of gene orientation [50], transcription [48, 114], and chromatin organizations [49, 114]. 

The *S* and *∆S* are basically equivalent to the ∆GC skew and B_I_ index. While the use of other indices of strand asymmetry in bacteria, such as the LDA prediction accuracy, the S/N of oligomer skew, and the GCSI, are limited due to their requirement for circular genomes, these indices should, in principle, be applicable to eukaryotic genomes and also to archaeal genomes with multiple origins [116, 117] by extracting a segment centered around the replication initiation sites, as in the calculation of *∆S*. Modification would be necessary for the LDA prediction accuracy due to the scarce nature of coding regions in mammals, and for the GCSI due to the N-shape of the strand skew rather than a discrete sine curve. However, the availability of multiple indices that assess the strength-of-strand asymmetry by alternative means would provide a stronger basis for these analyses. 

## SOFTWARE FOR THE CALCULATION OF STRAND BIAS MEASURES

There are a wide variety of software and web tools for the graphical analysis of GC skew and other compositional asymmetries, such as the Integrated Microbial Genomes (IMG) system provided by the Joint Genome Institute [118], and a comprehensive review is also available elsewhere [61]. Therefore, in this review, we focus on the software for the calculation of strand bias measures described thusfar. 

Analysis of strand asymmetry almost always requires the use of multiple indices or a combination of methods and careful selection or grouping of species or gene sets. Therefore, comprehensive analysis packages and environments are desirable for detailed studies. Two software packages are equipped with collections of tools for the study of compositional asymmetry – the SeqinR package in the R statistics language (http://www.r-project.org) [119] and the G-language Genome Analysis Environment (G-language GAE) in Perl [54, 120, 121]. Both packages support several formats of sequence flatfiles and can take advantage of the advanced statistical capabilities of programming environments. In this section, we illustrate the use of the G-language GAE to analyze strand bias because this software package contains almost all of the measures of strand bias that have been described thus far, and because the G-language GAE can be accessed *via *its web service interface [122]. The availability of the RESTful web service interface eliminates the requirement for software installation, setup, and maintenance, and users can access the service regardless of their platform. 

The web services of the G-language GAE can be used by specifying a URL in a browser according to a set of rules. The most basic syntax is the following:

(19) http://rest.g−language.org/[accession]/[program]/[option]=[value]/

where [accession] corresponds to the NCBI RefSeq accession number for the genome of interest, such as NC_000913 for *Escherichia coli* K12 MG1655, or an accession ID given by the G-language GAE after uploading a file at http://rest.g-language.org/upload/. The name of the program to use is [program], and multiple [option]=[value] pairs separated by a slash (/) can be appended for the configurable options. A list of programs, as well as the options that are related to the analysis of the replication-related strand bias, is shown in Table **2**, and a complete list of all programs in the G-language GAE and detailed documentation is available online at http://ws.g-language.org/gdoc/. For example, the calculation of the GCSI for the *E. coli* genome can be obtained by simply accessing the following URL

(20) http://rest.g−language.org/NC_000913/gcsi/

which immediately returns a GCSI of 0.096 with *SA* and *dist_norm_*. The options of programs can be configured to meet a variety of needs. Fig. (**3**) shows several examples of the GC skew analysis of *Escherichia coli*. Documentation for each of the programs, including the list of available options, can be viewed at

(21) http://rest.g−language.org/help/[program]/

There are also several other programs that are suited to the study of strand asymmetry in addition to those listed in Table **2**, such as the extraction of leading and lagging strand sequences based on the position of the origin in dOriC database [123] and the *dif* positions [124], or for the search of DnaA box, Ter, and iteron sites. More detailed documentations and live examples are available at http://www.g-language.org/wiki/rest. The G-language REST web service provides an intuitive and rapid method to use a variety of tools to study strand bias without the need for installation and setup. However, in order to automate an analysis pipeline that encompasses a number of tools applied to hundreds of genomes and to take advantage of the maximal efficiency of local computers, installation of the G-language GAE software is recommended. The latest software package and detailed documentation and tutorials are available at the project’s web site, http://www.g-language.org/. G-language GAE is free software licensed under the GNU General Public License version 2. 

## CONCLUSIONS

Quantitative measures of replication-related strand bias are necessary to quantify the different mutational pressures, to study complex biological causes of strand asymmetry, and to compare the degree of strand bias in genomes among the diverse clades of bacteria. Such studies require an extremely careful selection of the set of target species, genes, and genetic features to rule out potential biases due to pseudo-correlations. Therefore, the use of suitable measures of strand bias and possibly combining multiple methods based on different algorithms can be a critical part of the research. The availability of intuitive, RESTful services of these indices as part of the G-language GAE allows for quick, heuristic checking. Replication-related strand bias in eukaryotes has yet to be explored in detail, and measures of strand bias that are suitable for the analysis of eukaryotic origins would be an interesting area of research.

## Figures and Tables

**Fig. (1):**
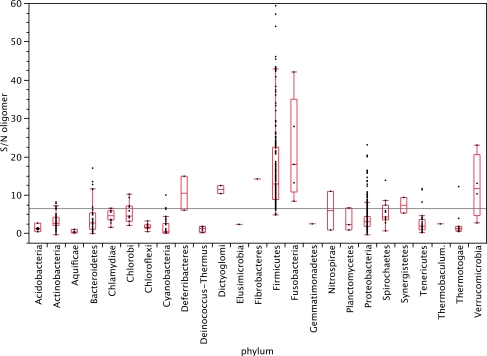
Distribution of the S/N of oligomer skew in different phyla of eubacteria. The degree of replication-associated strand bias shows a characteristic distribution among the bacterial phyla, and Firmicutes is one of the most highly biased groups. The majority of the Firmicutes contain the *polC* subunit of the DNA polymerase, which results in a high gene strand bias. In comparison, proteobacteria belong to a group with a moderate degree of strand bias.

**Fig. (2):**
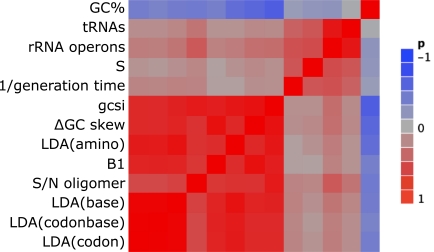
Clustering of the measures of strand bias and growth rate by Pearson correlations in 1083 bacterial genomes (only 254 genomes were used for 1/generation time). All strand bias measures are highly correlated (most above *r* > 0.70) and are also weakly correlated with properties related to the growth rate (*r* > 0.25).

**Fig. (3):**
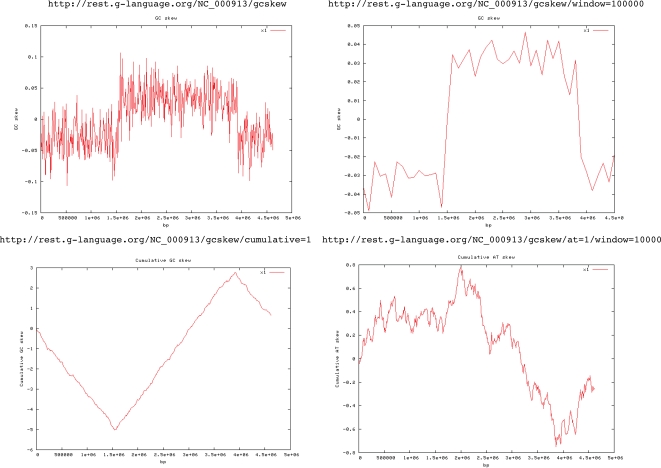
Examples of GC skew graphs using the G-language GAE web service. G-language REST web service can be easily used by typing a URL in a browser starting with http://rest.g-language.org/ followed by a set of commands. Here, the GC skew graphs of *Escherichia coli* (NC_000913) are visualized using different sets of options, such as normal or wider windows (window=100000), a cumulative graph (cumulative=1), and the AT skew at a window size of 10000 bp (at=1, window=10000). Neither registration nor setup is required to use these services, allowing the user to readily make use of these tools for their research.

**Table 1.** Summary of Strand Bias Measures

Index	Value range	Observing bias	Computation cost	*p-value*	Gene annotation	Replication origin and terminus	Circular genome
ΔGC skew	0 to 2	GC skew (GC3 only)	very low		required	required	
B_I_	0 to √2	GC and AT skews (GC3 only)	very low		required	required	
LDA prediction accuracy	0.5 to 1	gene skew	high	yes	required		required
S/N of oligomer skew	1 to ∞	oligomer skew	very high				required
GCSI	0 to 1	GC skew (all regions)	low	yes			required

^*^GC3 denotes third codon positions.

**Table 2.** Programs and Options for Strand Bias Analysis in the G-Language Genome Analysis Environment

Name	Option	Description
B1		BI index
B2		BII index
delta_gcskew	method=degenerate (default)	ΔGC skew using four-fold degenerate GC3
	method=gc3	ΔGC skew using GC3
	method=all	ΔGC skew using all bases
	at=1	ΔAT skew
	purine=1	ΔPurine skew
	keto=1	ΔKeto skew
gcsi		GC skew index
	at=1	AT skew index
	purine=1	Puine skew index
	keto=1	Keto skew index
lda_bias	variable=codon (default)	LDA prediction accuracy using 61 codons
	variable=base	LDA prediction accuracy using 4 bases
	variable=codonbase	LDA prediction accuracy using 12 bases/codon positions
	variable=amino	LDA prediction accuracy using 20 amino acids
gcskew		GC skew graph
	cumulative=1	cumulative GC skew graph
	at=1	AT skew graph
	purine=1	Purine skew graph
	keto=1	Keto skew graph
gcwin		GC content graph
	at=1	AT content graph
	purine=1	Purine content graph
	keto=1	Keto content graph
geneskew		gene skew graph
	cumulative=1	cumulative gene skew graph
	gc3=1	GC/AT/Purine/Keto skew graph in GC3 (specified with "base" option)
genomicskew		GC skew graph of coding/non-coding/GC3 regions
	at=1	AT skew graph of coding/non-coding/GC3 regions
dnawalk		DNA walk graph
find_ori_ter		origin / terminus prediction using cumulative GC skew
	at=1	origin / terminus prediction using cumulative AT skew
	purine=1	origin / terminus prediction using cumulative Purine skew
	keto=1	origin / terminus prediction using cumulative Keto skew
	filter=95	origin / terminus prediction using low-pass filtering with FFT
rep_ori_ter	gcskew=1	origin / terminus prediction using cumulative GC skew
	oriloc=1	origin / terminus prediction using Oriloc algorithm
	dbonly=1	origin / terminus prediction using dOriC and dif prediction data

^*^GC3 denotes third codon positions.
